# Determinants of Implementation of a Clinical Practice Guideline for Homeless Health

**DOI:** 10.3390/ijerph17217938

**Published:** 2020-10-29

**Authors:** Olivia Magwood, Amanda Hanemaayer, Ammar Saad, Ginetta Salvalaggio, Gary Bloch, Aliza Moledina, Nicole Pinto, Layla Ziha, Michael Geurguis, Alexandra Aliferis, Victoire Kpade, Neil Arya, Tim Aubry, Kevin Pottie

**Affiliations:** 1 Interdisciplinary School of Health Sciences, University of Ottawa, Ottawa, ON K1N 6N5, Canada; Omagwood@bruyere.org; 2 C.T. Lamont Primary Health Care Research Centre, Bruyère Research Institute, Ottawa, ON K1R 6M1, Canada; hanemaaa@uoguelph.ca (A.H.); ammar.saad@uottawa.ca (A.S.); Layla.ziha@uottawa.ca (L.Z.); 3 Department of Population Medicine, University of Guelph, Guelph, ON N1G 2W1, Canada; nicole.pinto11@gmail.com; 4 School of Epidemiology and Public Health, University of Ottawa, Ottawa, ON K1N 6N5, Canada; 5 Department of Family Medicine, University of Alberta, Edmonton, AB T6G 2R3, Canada; ginetta@ualberta.ca; 6 Department of Family and Community Medicine, St. Michael’s Hospital, Toronto, ON M5C 2T2, Canada; gary.bloch@utoronto.ca; 7 Faculty of Medicine, University of Toronto, Toronto, ON M5S 1A8, Canada; 8 Inner City Health Associates, Toronto, ON M5C 1K6, Canada; 9 Faculty of Medicine, University of Ottawa, Ottawa, ON K1N 6N5, Canada; almoledina@toh.ca; 10 Faculty of Health Sciences, University of Ottawa, Ottawa, ON K1N 6N5, Canada; 11 Department of Health Sciences, Wilfrid Laurier University, Waterloo, ON N2L 3C5, Canada; geur4550@mylaurier.ca (M.G.); narya@uwaterloo.ca (N.A.); 12 Michael C. DeGroote School of Medicine, McMaster University, Hamilton, ON L8S 4L8, Canada; aaliferis@gmail.com; 13 Department of Medicine, McGill University, Montreal, QC H3A 0G4, Canada; victoire.kpade@mail.mcgill.ca; 14 Department of Family Medicine, McMaster University, Hamilton, ON L8S 4L8, Canada; 15 School of Psychology & Centre for Research on Educational and Community Services, University of Ottawa, Ottawa, ON K1N 6N5, Canada; taubry@uottawa.ca; 16 Department of Family Medicine, University of Ottawa, Ottawa, ON K1N 6N5, Canada

**Keywords:** guideline implementation, knowledge translation, determinants of evidence uptake, homelessness, GRADE FACE Survey, health equity

## Abstract

Clinical practice guidelines can improve the clinical and social care for marginalized populations, thereby improving health equity. The aim of this study is to identify determinants of guideline implementation from the perspective of patients and practitioner stakeholders for a homeless health guideline. We completed a mixed-method study to identify determinants of equitable implementation of homeless health guidelines, focusing on the Grading of Recommendations Assessment, Development and Evaluation Feasibility, Acceptability, Cost, and Equity Survey (GRADE-FACE) health equity implementation outcomes. The study included a survey and framework analysis. Eighty-eight stakeholders, including practitioners and 16 persons with lived experience of homelessness, participated in the study. Most participants favourably rated the drafted recommendations’ priority status, feasibility, acceptability, cost, equity impact, and intent-to-implement. Qualitative analysis uncovered stakeholder concerns and perceptions regarding “fragmented services”. Practitioners were reluctant to care for persons with lived experience of homelessness, suggesting that associated social stigma serves as a barrier for this population to access healthcare. Participants called for improved “training of practitioners” to increase knowledge of patient needs and preferences. We identified several knowledge translation strategies that may improve implementation of guidelines for marginalized populations. Such strategies should be considered by other guideline development groups who aim to improve health outcomes in the context of limited and fragmented resources, stigma, and need for advocacy.

## 1. Introduction

People experiencing homelessness face health inequities including high rates of preventable all-cause mortality [1], and treatable morbidities such as infectious diseases and chronic health conditions [2]. The origins of these health inequities are complex and involve individual and system-level factors such as cultural and structural violence [3,4]. Until recently, no evidence-based guidelines for populations experiencing homelessness existed. The recent development of a clinical practice guideline for this population, termed the ”Homeless Health Guideline” [5], may help to address upstream factors that lead to disparities, thereby addressing an array of social and health concerns while simultaneously empowering homeless patients in their own self-determination [6]. The guideline introduces an upstream approach where permanent supportive housing is viewed as a core intervention, along with other clinical and community interventions (see Table 1), which will improve the health and social outcomes of individuals with lived experience of homelessness.

The Homeless Health Guideline was developed using an evidence-based approach, drawing on the evidence from seven systematic reviews [7,8,9,10,11,12,13]. There are opportunities and challenges that arise when bringing new clinical practice guidelines into policy and practice [14]. Previous guidelines have highlighted that health equity implementation concerns exist among marginalized communities [15,16]. Patient and practitioner perceptions and concerns may create significant barriers to implementation. An understanding of determinants of implementation of the Homeless Health Guideline is necessary to identify promising implementation strategies. ”Determinants of implementation” are factors that might be perceived to prevent or enable the implementation of a clinical practice guideline. Such factors have also been referred to as barriers and enablers, barriers and facilitators, problems and needs, or disincentives and incentives [17,18]. These barriers may contribute to ongoing health inequities faced by this population. It is important to understand these perceptions and identify promising implementation strategies to enhance the adoption, implementation, and sustainability of the guideline [6,19,20]. The Grading of Recommendations Assessment, Development and Evaluation Feasibility, Acceptability, Cost, and Equity Survey (GRADE FACE) is one tool that has been developed to assess determinants of evidence-based guideline implementation and promote health equity [21].

This study aims to address the following exploratory research question: What are the perceived determinants of the implementation of the Homeless Health Guideline?

## 2. Materials and Methods

### 2.1. Design

We conducted a pragmatic mixed method survey among key stakeholders and individuals with lived experience of homelessness which included close-ended and open-ended narrative questions. We chose to employ mixed methods as it combines the generalization offered by quantitative research with the depth of detailed understanding offered by qualitative research. We prioritized the qualitative data collection and analysis, a decision which was influenced by the purpose of the study to identify and explain the determinants of guideline implementation. We followed the GRADE-FACE Survey [21] and added questions that aimed to capture and learn from the experiences of the respondents. FACE is an implementation survey that makes it possible to assess and present complex stakeholder perceptions in a useful manner that may help initiate knowledge mobilization among diverse stakeholders [21]. We report our findings according to the consolidated criteria for reporting qualitative research (COREQ) checklist (see Additional File 1).

### 2.2. Research Team and Reflexivity

We assembled a multidisciplinary research team with expertise in primary care, marginalized populations, and research methodologies. A subset of our research team was responsible for data collection and engaged with study participants: two clinician-investigators (KP, NA) whose work includes homeless populations; one research associate (OM) with experience in qualitative research; and seven medical (AA, VK), graduate (AH, AS, NP), and undergraduate (LZ, MG) students with prior research experience. The group consisted of four males and six females. Four group members self-identified as a visible minority. No members identified as Indigenous or as a sex and/or gender minority. Two members had lived experience of living in a low-income household, and one member had lived experience of homelessness or vulnerable housing. Previous professional relationships existed between the research team and some survey respondents.

### 2.3. Participants and Setting

Participants were invited to participate in the study if they were practitioners or service providers working with populations experiencing homelessness in Canada, or if they were an individual with lived experience of homelessness. Stakeholders were contacted using direct email to members of the Homeless Health Research Network [22], and snowball sampling whereby network members were asked to identify additional potential respondents. Persons with lived experience were selected by staff at our partner non-governmental organizations (NGOs) through face-to-face invitations to participate. To protect participant confidentiality, we do not report specific locations. Participants were recruited until data saturation was achieved—i.e., until no new relevant knowledge was being obtained from new participants.

### 2.4. Survey

The Grading of Recommendations, Assessment, Development and Evaluation (GRADE) working group developed the Feasibility, Acceptability, Cost and Equity (FACE) Implementation Survey. This survey aims to capture the perceptions of respondents regarding the following six determinants of implementation in order to identify promising implementation strategies [21]:

- Priority: Determines whether the intervention considered is serious or urgent, whether the consequences of inaction are severe, or if the intervention is a recognized priority [21].
- Feasibility: The extent to which a new practice can be successfully carried out within a given setting. Feasibility of the recommendation is determined by considering whether the proposed recommendation is sustainable and the barriers to implementing the recommendation can be addressed [21,23,24].
- Acceptability: The perception among implementation stakeholders that a given practice is appropriate, agreeable, palatable, or satisfactory [21,23,24].
- Cost: The cost impact of an implementation effort [23,24].
- Equity: Whether groups or setting may be disadvantaged by interventions [25].
- Intent-to-implement: The intention, initial decision, or action to try to employ an evidence-based practice [23,24].

The survey (see Additional File 2) covered the five interventions outlined in Table 1. Stakeholders, including practitioners, service providers, and persons with lived experience, were first asked to prioritize the interventions. They were also asked about each of the recommendations to gain insight into perceptions regarding feasibility, acceptability, cost, and impact on health equity. They were also asked about their intent to implement the recommendations. The survey ended with four open-ended questions to further contextualize findings.

### 2.5. Ethics Approval and Consent to Participate

This study was approved by the Bruyère Continuing Care Research Ethics Board (#M16-18-043) of the Bruyère Research Institute, Ottawa, ON, Canada.

All participants provided informed consent for survey and interviews.

### 2.6. Data Collection

Based on the findings of a scoping review that outlined approaches to engaging with marginalized populations [26], we identified a need to consider oral (narrative) methods and plain language approaches that may mitigate power dynamics between the researcher and participant. Therefore, the survey was available online and on paper for all stakeholders, and persons with lived experience of homeless were offered the opportunity to complete the survey verbally with a member of the research team.

The survey was available to stakeholders online for a period of 73 days between April and June 2019. Persons with lived experience of homelessness completed the survey verbally between May and July 2019. Verbally administered surveys occurred within NGOs or shelters serving homeless populations. No one other than the individual participant and members of the research team were present during the survey except in one case when requested by the participant. Surveys were scheduled for 30–45 min. The responses were audio-recorded, de-identified, and transcribed verbatim. Transcriptions were cross-checked against the audio recordings for accuracy. Surveys occurring in French were translated into English for analysis. Members of the research team kept field notes during the surveys to verify transcription accuracy. Due to the transient nature of participant locations, transcripts could not be returned to participants for comment. Participants received an honorarium in the form of a CAD 30 gift card for their participation.

### 2.7. Analysis

We analyzed survey data from close-ended questions using descriptive statistics and tabulated our results. We elected not to perform subgroup analyses due to the sample size, but present disaggregated data in Additional File 3.

We used the ”best fit” framework method as a systematic and flexible approach to analyzing the qualitative data from open-ended questions [27,28,29]. “Best fit” framework synthesis requires identification of a relevant framework for health behaviours, which is then reduced to its key elements. These key elements form the initial themes of the framework analysis. Framework-based synthesis using the ”best fit” method is a highly pragmatic and useful strategy for a range of urgent policy questions [30], whose use is supported in guideline development literature [31]. Framework analysis is a five-stage process that includes familiarization with the data, identifying a thematic framework, indexing (applying the framework), charting and mapping, and interpretation [32]. We selected the Theoretical Domains Framework (TDF) based on the specificity of its constructs for the analysis of implementation-related behaviours. This framework provides a theoretical lens for understanding the processes involved in translating evidence-based recommendations into healthcare practice, through which facilitators and barriers relevant to implementation are categorized across cognitive, affective, social, and environmental domains [33]. Developed through a multidisciplinary consensus approach and subsequent validation, TDF consolidates overlapping behavioural theories into 14 domains encompassing 84 theoretical constructs (see Additional File 4) [33].

TDF domains were adopted as themes for qualitative analysis of transcribed surveys. After ensuring familiarity with the data, data were coded independently in duplicate by AH and LZ into the most relevant TDF domains. Coding was verified by OM and KP. Discrepancies were resolved by means of discussion. We used participant confirmation (“member checking”) by three individuals who provide services to homeless populations to enhance the credibility of our findings. We mapped the frequency of message units per TDF domain to the FACE criteria and produced a heat map to identify the key findings regarding feasibility, acceptability, cost, equity, and intent-to-implement.

## 3. Results

### 3.1. Participants

A total of 88 health and social service providers and persons with lived experience of homelessness across six Canadian provinces completed the FACE survey. This included 76 participants who completed the survey online and 16 in-person interviews. The response rate was 85.22%. The FACE survey took 20–45 min to complete. Table 2 presents participant characteristics. The majority of participants were between 31 and 50 years of age, 15.9% were younger than 30 years of age, and 12.5% were older than 61 years of age. The majority (55.7%) of participants identified their gender as female. Most participants resided in Ontario (60.2%), Alberta (18.2%), and Quebec (13.6%). English was the primary language spoken by 84.1% of participants, and 36% of participants worked as primary healthcare providers. Sixteen participants (18.2%) reported lived experience of homelessness. Twenty-five participants (28.4%) reported more than 11 years of involvement in the field of homeless health and research, followed by 19 participants (21.6%) who reported 6–10 years of experience.

**Figure 1:**
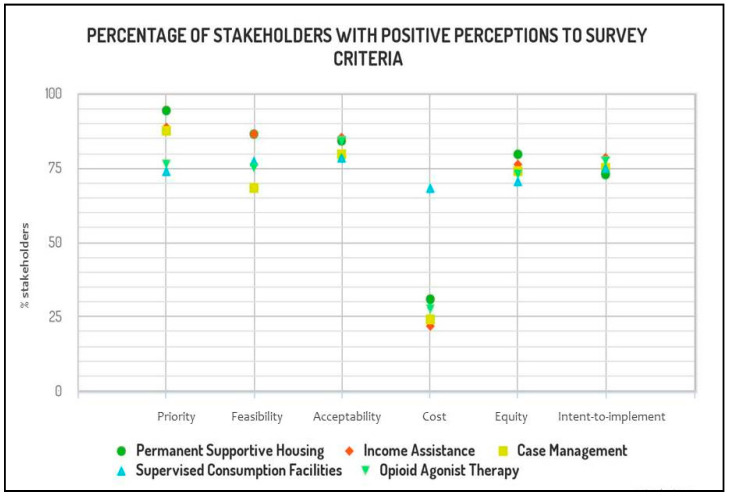
Positive stakeholder trends on five recommendations presented as the percentage of participants who reported that the intervention at hand is (or probably is) a priority, and that implementing such intervention is (or probably is) feasible, acceptable, has (or probably has) a positive impact on health equity, and brings moderate to large savings as opposed to negligible savings or moderate or large costs.

The perceptions of our 88 respondents on the feasibility, acceptability, impact on cost and health equity, as well as their intention to implement the recommendations of the Homeless Health Guideline are reported using the FACE scale—“yes, probably yes, no, probably no, varies, don’t know”. To descriptively report our findings, we collapsed our ordinal response options to reflect the overall positive or negative trends in perceptions (see Figure 1 and Table 3).

**Figure 2:**
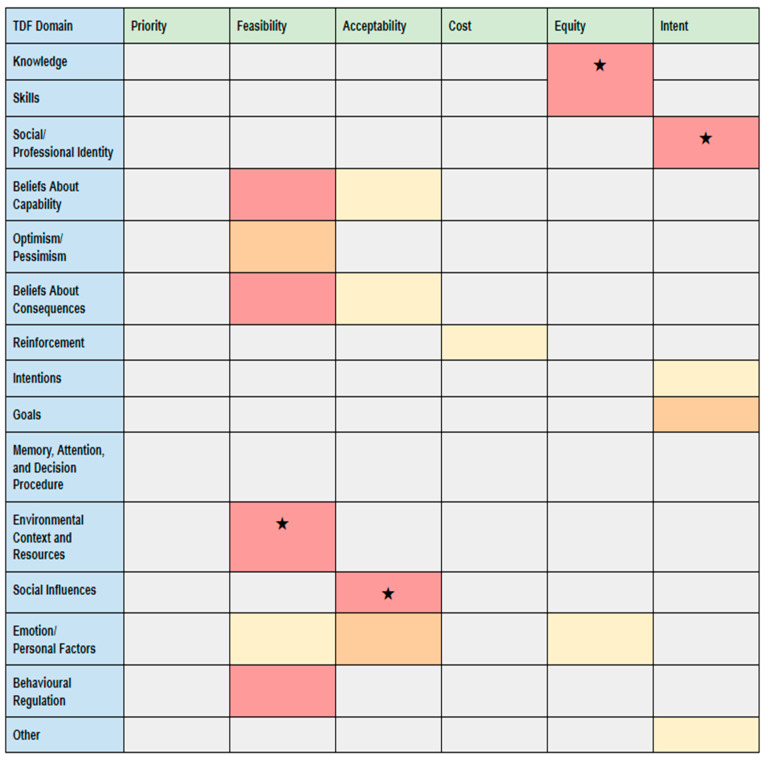
Heat map of the Theoretical Domains Framework (TDF) and FACE criteria. Legend: 0 comments, 1–2 comments, 3–4 comments, 5 + comments; ★ key finding (highest concentration of message units).

Our heat map (see Figure 2) shows the frequency of message units per TDF domain according to each of the FACE criteria, where darker colours indicate a higher concentration of message units and star icons (★) indicate key findings. We identified key findings regarding feasibility, acceptability, cost, equity, and intent-to-implement. These key findings related to environmental context and resources, social influences, practitioner knowledge and skills, and professional identity.

### 3.2. Selecting Priority Interventions

The majority of our respondents perceived permanent supportive housing (94.3%), income assistance (88.6%), and case management (87.5%) as priority interventions. The extent to which they perceived supervised consumption facilities (73.9%) and opioid agonist therapy (76.1%) as a priority was lower. Twelve participants (13.6%) responded “it varies” when asked if supervised consumption facilities were a priority, while 11 (12.5%) had this response regarding opioid agonist therapy.

### 3.3. Feasibility

Implementation of the recommendations for permanent supportive housing was perceived to be feasible by a large majority (86.3%) of our respondents. The same proportion (86.3%) perceived the recommendation on the provision of income assistance to be feasible as well. Notably, the recommendation on case management generated conflicting opinions: 68.1% believed that this recommendation is feasible, while 13.6% refuted its feasibility, and 9% responded that the feasibility was variable. The recommendations for supervised consumption facilities and opioid agonist therapy showed similar patterns of participant response: 77.2% of respondents perceived the recommendation for supervised consumption facilities as feasible, while 76.1% agreed with that opioid agonist therapy is feasible as a recommendation. Despite favourable perceptions, respondents also highlighted several barriers to implementation in their qualitative responses, outlined below.

#### Theme 1: Insufficient Resources (TDF: Environmental Context and Resources)

Service providers and individuals with lived experience of homelessness expressed how resource availability, proximity of services to supportive housing, and organizational culture can affect the implementation of these guidelines. Participants highlighted limited availability of resources, including affordable and permanent supportive housing, case management services, and harm reduction programs, as an important barrier to guideline implementation, particularly in rural settings. Regarding the availability of permanent supportive housing, one clinician stated:

*“vulnerably housed people end up waiting a long time for housing, or may never get housed. In many cases, we end up putting people into inappropriate housing models without support, just to get them off the street.”*

The insufficiency of resources may be reasonably understood to not only limit the implementation of longitudinal interventions for homeless populations but further to compromise the quality of housing experienced by those who are homeless or vulnerably housed. A participant with lived experience of homelessness described the under-resourced shelter experience, explaining that:

*“if you don’t live here, you don’t understand, but it’s hell. It’s hell every day. It’s dirty. You have to almost beg for toilet paper.”*

Participants also raised the issue of administrative difficulties faced by homeless individuals that preclude them from accessing housing even had it been available. One individual described:

*“I was homeless from the age of 13 to about 26. I never stayed in a shelter, and I was never housed. So, I slept outside, I couch surfed… I know I would have really benefited from supportive housing—one that came with no strings attached, no hoops to jump through, just a safe place to go… would have probably saved my life a lot sooner.”*

*“everybody wants housing, and in order to get housing through a shelter, you need six months consecutive stay [before] they can even do a housing [intake assessment], in which you’re on the waiting list for ten years”*

Recognizing the importance of housing that has embedded support for homeless individuals with concurrent mental illness or substance use, many participants also raised concerns about the lack of proximity of health and social services to housing arrangements intended for those experiencing homelessness. For instance, one participant with lived experience of homelessness emphasized:

*“when you have [services] scattered across the city, the Housing First sometimes doesn’t work, because the client isn’t able to access supportive services that go hand-in-hand with [it].”*

A clinician similarly reported that “buildings are not zoned for assisted living,” thus making necessary services inaccessible to homeless individuals placed in permanent housing. Respondents consistently corroborated that the proximity of healthcare, social services, and harm reduction programs, including supervised consumption facilities, may dictate the effectiveness of permanent supportive housing in promoting housing retention. This is particularly true for those requiring higher intensity care. Furthermore, several clinicians emphasized the importance of primary care clinics as a low-barrier setting capable of coordinating complex care needs:

*“Practitioners need not only to be aware of the needs of this population, but also need to be able to provide the majority of care required, including specialist mental health and addiction care, assistance with the social determinants of health, and a focus of reducing the number of organizations and institutions involved in the individual’s care.”*

*“Separating mental health/addictions treatment and support from the locus of primary care reduces effectiveness, increases costs, and reduces client acceptability. Mental health treatment needs to be onsite with primary care.”*

### 3.4. Acceptability

There were similar trends in responses for stakeholder acceptability compared to perceptions regarding feasibility of implementation. Seventy-five respondents (85.2%) reported the recommendations for income assistance to be acceptable, and seventy-four respondents (84%) reported the same about permanent supportive housing as well as opioid agonist therapy (84%). Seventy respondents (79.5%) believed implementing case management was acceptable (79.5%), and 69 respondents (78.4%) believed the same about supervised consumption facilities. However, several persons with lived experience of homelessness described how stigma, discrimination, and other societal influences can limit the acceptability of these services.

#### Theme 2: Stigma and Discrimination (TDF: Social Influences)

Stigma attached to the condition of homelessness was recognized as an important barrier to care for homeless populations. This was particularly noted in cases of intersectional discrimination, including concomitant mental illness or substance use, and social alienation. A case manager, when speaking about her experience connecting homeless and vulnerably housed individuals with active substance use to healthcare, stated that “select few clinics that we work with will take our clients”. Another case manager corroborated that when attending appointments with a homeless client, “the doctor treats them like a second class citizen because they happen to smell or they’re homeless”. A case worker similarly explained that:

*“we have a couple great clinics that work with our clients, that’ll take them however they are, and see them even if they’re late, make spots for them… but it’d be nice if there was more access to family doctors, so somebody could have a stable family doctor and a treatment plan going forward with their health.”*

This sentiment was also expressed by a person with lived experience of homelessness:

*“For the physicians, […] there needs to be more training around stigma. Maybe some education around how to not treat people like they don’t have value might be a good idea.”*

Discrimination associated with substance use was also noted as a barrier for accessing permanent supportive housing. Speaking about abstinence-based housing programs, one participant with lived experience noted that there is “stigma of, ‘You’re still using? You don’t deserve [housing]”. Similar concerns were raised regarding the attitudes of other social service providers, including those responsible for approving income assistance for individuals struggling financially. Speaking about social welfare administrators, one participant recounted: “They’re condescending… they treat everybody like we’re stealing from them”. Accordingly, when faced with discrimination by health and social service providers, stigma may undermine the quality of care received by individuals experiencing homelessness, from the point of reception into care and throughout the duration of treatment.

On the contrary, the presence of social support was recognized as a catalyst for the improvement of health outcomes and transition out of homelessness for individuals with housing instability. One individual, speaking about their experience with permanent supportive housing, stated that: “There’s a community network here that doesn’t exist in the shelter system, and that’s one where you can feel safe”. Several other participants with lived experience of homelessness emphasized that the presence of a committed case manager or social worker was imperative to their recovery and wellbeing. Recounting her struggle with overcoming substance use, one individual noted that it was her “case manager that gave [her] that hope to stay clean”.

### 3.5. Costs and Savings

When asked about the impact of the recommendations on costs and savings, conflicting perspectives emerged amongst survey participants (see Table 2). Participants perceived moderate cost for all interventions except supervised consumption facilities, which were perceived as cost-saving.

Few participants provided comments on the impact of the recommendations on costs and savings, although one noted that “intuitively, efforts to reduce homelessness should produce system cost savings in healthcare and the justice system”. However, several participants highlighted significant bottlenecks to implementation of these recommendations, particularly around the lack of investments towards affordable housing and the human resources needed to provide case management and other supports.

### 3.6. Health Equity

The majority of respondents perceived the recommendations for permanent supportive housing (79.5%), income assistance (76.1%), case management (73.8%), supervised consumption facilities (70.4%), and opioid agonist therapy (72.4%) to have a positive impact on health equity. Only eight respondents (9%) reported that they foresaw reduced health equity resulting from the permanent supportive housing recommendations. Similarly, eight respondents (9%) reported reduced health equity for income assistance recommendations. Respondents outlined important educational needs to improve health equity.

#### Theme 3: Capacity Building for Healthcare Providers (TDF: Knowledge and Skills)

Awareness of the circumstances experienced by homeless individuals, and the specific needs associated with varying levels of homeless chronicity, was emphasized as a prerequisite for quality healthcare and social service provision. As noted by one participant with lived experience, and corroborated by various other participants across all stakeholder categories, service providers “should be educated on the needs of the homeless—what is needed, the procedures, and the availability of things so [they] can help people”. The participant noted disappointment in how service providers often leave the role of “expert” to their patients. Describing their experience with providers, one individual explained how:

*“they really… they don’t know… like I know more about it than they do, you know. And that shouldn’t be. I should not be the expert.”*

One primary care practitioner expressed that “homeless people often receive less help from those who are unfamiliar with the challenges that homeless people face’’. This further highlights the need for both technical and experiential training of service providers and the use of peer support providers if the objective of integrating longitudinal and sustainable interventions for homeless health into the locus of primary care is to be realized. One participant aptly noted that, in its current state, “the very system itself works against the people who need the services the most”.

Some participants also expressed concerns about the capacity of healthcare providers to promote substance use interventions. One physician noted:

“*[…] The majority of docs aren’t comfortable prescribing OAT [Opioid Agonist Therapy] or they don’t know how to recognize opioid use disorder in their practice. These patients then end up having to access “specialty” clinics when they would do best in the care of their primary care provider.*”

However, others recognized that capacity is being built for harm reduction approaches to substance use disorders:

*“We are working on training family physicians to do both [primary care and addiction treatment], but this will take time, and is likely also constrained by the fee for service billing model we have in place, which does not support family docs to do both.”*

### 3.7. Intent-to-Implement

The majority of respondents responded that they intended to implement the recommendations on permanent supportive housing (72.7%), income assistance (78.4%), case management (75%), supervised consumption facilities (75%), and opioid agonist therapy (77.2%). Only six respondents (6.8%) did not intend to recommend case management to their clients, and only five (5.6%) did not intend to recommend supervised consumption facilities.

#### Theme 4: Comprehensive Primary Care (TDF: Professional Role and Identity)

While many participants recognized the benefits of longitudinal interventions, various health and social service providers anticipated difficulty implementing the guidelines in their current practice. Some providers questioned the approach of addressing social determinants of health in a primary care setting, with one physician saying “Allied health professionals do not always agree with their role in this area of ‘prevention’”. However, many clinicians held the opposite belief and favoured primary care as the main location for this type of prevention and care coordination:

*“Insufficient primary care capacity is the biggest ongoing barrier--all the other services function much better/more efficiently when grounded in a foundation of skilled primary care.”*

Many clinicians also expressed frustration that primary care clinics currently have insufficient capacity to meet the needs of vulnerable populations as they try to navigate a complex process:

*“There is lack of transparency in the process [regarding housing] and it can be time consuming as a health care provider to figure out how and to whom to advocate due to fragmented services.”*

Others recommended the expansion of the multidisciplinary primary care team to better address social determinants of health in a primary care setting:

*“Due to the often complicated nature of applying and accessing social determinants of health resources, and the amount of time and more specialized expertise required to help patients figure out which resources they are eligible for and submit applications for, it would be even more beneficial to have more access to social workers who can help patients access these resources.”*

## 4. Discussion

In this study, we employed a mixed method approach to highlight the perceptions of practitioners and people experiencing homelessness around the determinants of implementing a clinical guideline for homeless health. This study contributes to a growing body of international evidence on effective interventions for populations experiencing homelessness [7,8,9,10,11,12,13] and implementation tools [21,34,35]; Furthermore, our study has a unique focus on the health equity concerns of guideline implementation, which is often excluded from existing taxonomies of implementation outcomes [24]. Understanding the perceptions of stakeholders and end-users around contextual and population-specific implementation barriers and enablers will allow us to better understand and put forward effective and successful implementation initiatives following the development of our guideline. The quantitative and qualitative results of the FACE survey identified concerns regarding feasibility, acceptability, and impact on patients’ health equity as well as incurred costs and projected savings.

The perceived limited availability of fragmented local resources such as permanent supportive housing was identified as an important feasibility concern and constraint to implement our recommendations in a local context. Indeed, limited availability of adequate and affordable housing is well recognized as a structural factor influencing societal predispositions to homelessness [2]. Without coordinated community-based and primary care programs delivered simultaneously with permanent housing, addressing social and health conditions remains challenging. Integrated care has, therefore, emerged as an essential approach to mitigate the fragmented care provided to individuals experiencing homelessness. Policy makers, primary healthcare practitioners, public health, and allied health professionals must work alongside one another to build capacity for providing adequate and equitable healthcare, and to maintain a suitable environment and medical home for people experiencing homelessness.

Discrimination towards populations experiencing homelessness was found to be a significant barrier to the implementation of our guideline. Such discrimination was reported predominantly by people with lived experience of homelessness. Notably, it is not the recommendations themselves that lead to discrimination and low acceptability, but rather the stigmatizing context in which they would be delivered. Stigma experienced by homeless persons can be propagated through interactions with health care practitioners, thus perpetuating power imbalances, negative stereotypes, and inequalities [36] associated with structural violence [37]. Stigma experienced by people experiencing homelessness in the context of receiving healthcare services could deter willingness to seek healthcare resources and negatively impact the depth of the relationship with healthcare providers [38,39]. Evidence shows that persons experiencing homelessness appreciate a clinical environment that emphasizes mutual trust, respect, and personal safety [10,39]. It is, therefore, imperative that any person who has direct contact and interaction with homeless persons in these contexts be trained in stigma or trauma-informed practices [40]. Essential components include trauma awareness, an emphasis on safety, and opportunities to build control [41]. The use of peer support workers in primary care clinics can help to counter stigma and welcome patients with lived experience into primary care practices [42].

Lack of knowledge among practitioners emerged as a major barrier to achieving health equity through guideline implementation. This finding suggests that several knowledge translation activities may have the potential to improve implementation. For example, our findings suggest that homeless-specific training for health professionals may be critical to improving care received by this vulnerable population. Health professional trainees could benefit from Community Service Learning programs which provide exposure to the health issues of marginalized populations and opportunities to practice skills such as communication, cultural safety, a community-grounded perspective, and advocacy for health [43]. At the same time, it can be expected that relying on training has its limitations and only certain primary care physicians will be open to serving people with lived experience [39].

The findings of this study provided a rich and comprehensive understanding of the challenges associated with implementing the Homeless Health Guideline [5]. This guideline is, to our knowledge, the first evidence-based clinical guideline for homeless health, and as such our study contributes novel barriers and facilitators to implementation. Although some of the challenges are unique to our recommendations, many could prove useful for guidelines targeting other marginalized populations. For example, guidelines for refugees and migrants have highlighted the importance of local context, availability of screening and treatment, and practitioner training to improve guideline implementation [15,44]. Moreover, we used the FACE Framework, endorsed by the GRADE working group, to engage stakeholders and highlight their perceptions on the feasibility, acceptability, implications on cost, and intent-to-implement of the recommendations. These criteria are recognized as conceptual implementation outcomes [24]. We additionally decided to study equity as an implementation criterion because we recognized its importance when developing and implementing guidelines among vulnerable populations [45], and in keeping with the belief that interventions promoting equity may be associated with increased sustainability [46].

Our work is not without limitations. Firstly, survey respondents were selected from our established networks, and thus they are more likely to be aware of the needs and resources available for this population. It remains unknown how a less aware practitioner would perceive this guideline. Furthermore, our work did not take into account the specific determinants of implementing this guideline in Indigenous contexts, as Indigenous homelessness is being researched in parallel by an Indigenous-specific research team [47]. Similarly, we considered the provinces in which our participants lived, but did not ask participants to disclose whether they lived in an urban or rural environment. It is expected that the availability of resources, and thus the feasibility of the recommendations, would differ in these circumstances. Thirdly, although we achieved data saturation, the transient nature of the homeless population resulted in challenges scheduling and completing interviews. It is possible that the verbal administration of the survey and opportunities for the research team to prompt the participant for further details resulted in qualitative findings from people experiencing homelessness being over-represented in our study. We did not establish any a priori hypotheses to detect any differences in stakeholder perceptions to each of the FACE criteria based on their occupation, location, or lived experience. To note, the data presented in this study represent the pre-implementation perceptions and accounts of participants and, as such, may not represent the guidelines’ actual influences on practice. Finally, policy makers were not included in our cohort of stakeholders, and thus, their missing perceptions may have provided more insight into the feasibility concerns raised by other stakeholders, and brought about policy and system-level change in the implementation of our guideline.

## 5. Conclusions

The Homeless Health Guideline is a promising advance in defining and improving the consistency and quality of care for people experiencing homelessness, combining rigorous evidence, clinical expertise, and patient preferences. In the context of clinical practice guidelines for marginalized populations, significant concerns may persist around the availability and accessibility of resources. Our study provides evidence for intended behaviour change, but variability in team capacity and outcome expectations suggest that ongoing research and knowledge translation strategies will be needed for sustainable implementation. The findings from this study will be used to develop a targeted, evidence- and theory-informed knowledge translation plan to support primary care for persons experiencing homelessness. Such approaches should be considered by other guideline development groups who aim to improve the health outcomes of marginalized populations.

## Figures and Tables

**Table 1:** Interventions and recommendations to improve the health of persons experiencing homelessness [5].

Intervention	Definition	Recommendations
Permanent supportive housing	Long-term housing in the community with no set pre-conditions to access it. Housing is combined with individualized supportive services that are tailored to participants’ needs and choices (e.g., assertive community treatment (ACT) or intensive case management (ICM)).	Identify homelessness or housing vulnerability and willingness to consider housing interventions. Ensure access for homeless or vulnerably housed individuals to local housing coordinator or case manager (i.e., dial 211 or via a social worker) for immediate link to permanent supportive housing and coordinated access system.
Income assistance	Benefits and programs that improve socioeconomic position. These include assistance that directly increases disposable income and programs that help with cost reduction to improve access to basic living necessities.	Identify income insecurity. Assist individuals with income insecurity to identify income support resources and access income.
Case management	Standard Case Management allows for the provision of a wide range of services with the goal of helping the client maintain good health and social relationships. Intensive Case Management (ICM) offers the support of a case manager that brokers access to an array of services. The case manager can be available for up to 12 h per day, 7 days a week and often has a caseload of 15–20 service users. Assertive Community Treatment (ACT) offers team-based care by a multidisciplinary group of healthcare workers in the community. This team is available 24 h per day, 7 days a week. Critical Time Intervention (CTI) supports continuity of care for service users during times of transition. It is administered by a CTI worker and is a time-limited service, usually lasting 6–9 months.	Identify history of severe mental illness, such as psychotic or mood and anxiety disorders associated with significant disability, substance use or multiple/complex health needs. Ensure access to local community mental health programs, psychiatric services for assessment, and linkage to intensive case management (ICM), assertive community treatment (ACT), or critical time intervention (CTI) where available.
Pharmacological interventions for substance use	Pharmacological interventions for opioid use disorder: Opioid therapy medications, such as methadone, buprenorphine, and buprenorphine/naloxone. Pharmacologic agents for reversal of opioid overdose: Opioid antagonist administered intravenously or intranasally—e.g., naloxone.	Identify opioid use disorder. Ensure access within primary care or via an addiction specialist to opioid agonist therapy (OAT), potentially in collaboration with public health or community health centre for linkage to pharmacological interventions.
Harm reduction interventions for substance use	Supervised consumption facilities (SCFs): facilities where people who use substances can consume pre-obtained substances under supervision. Managed alcohol programs (MAPs): shelter, medical assistance, social services and the provision of regulated alcohol to help residents cope with severe alcohol use disorder	Identify, during history or physical examination, problematic substance use including alcohol or other drugs. Identify the most appropriate approach or refer to local addiction and harm reduction/prevention services (e.g., supervised consumption facilities, managed alcohol programs) via appropriate local resources such as public health or community health centre/CLSC

**Table 2:** Participant demographics.

Characteristic	N	%
**Age**		
<30 years	14	15.9
31–40 years	24	27.3
41–50 years	23	26.1
51–60 years	14	15.9
61+ years	11	12.5
Missing	2	2.27
**Gender**		
Male	38	43.2
Female	49	55.7
Missing	1	1.14
**Province**		
British Columbia	5	5.68
Alberta	16	18.2
Ontario	53	60.2
Quebec	12	13.6
Nova Scotia	1	1.14
Prince Edward Island	1	1.14
Missing	0	0.00
**First Language**		
English	74	84.1
French	7	7.95
Other ^a^	5	5.68
Not reported	2	2.27
**Profession**		
Primary care provider	32	36.4
Specialist physician	10	11.4
Registered nurse	4	4.55
Public health expert	1	1.14
Social worker	1	1.14
Homelessness health researcher	10	11.4
Community health advocate	0	0.00
I am or have been homeless ^b^	16	18.2
Other ^c^	13	14.8
**Length of involvement in homelessness research or programs**		
<2 years	12	13.6
2–5 years	10	11.4
6–10 years	19	21.6
11+ years	25	28.4
Not applicable	22	25.0
Missing	0	0.00

^a^. Other first languages include: Hindi, Mandarin, Czech, Dutch, and Michif. ^b^. Includes being vulnerably housed, defined as living in poor quality, temporary, or precarious type of housing, including single room hotels, shelters, or rooming houses. ^c^. Other professions included: licensed nurse practitioner, unit clerk, CEO of homeless-serving organization, clinical manager, and medical student.

**Table 3:** Perception trends among *n* = 88 respondents to the Feasibility, Acceptability, Cost, and Equity (FACE) survey.

**Is the intervention a priority?**					
	**Permanent Supportive Housing** **n (%)**	**Income Assistance** **n (%)**	**Case Management** **n (%)**	**Supervised Consumption Facilities** **n (%)**	**Opioid Agonist Therapy** **n (%)**
Positive perceptions *	83 (94.32)	78 (88.64)	77 (87.5)	65 (73.86)	67 (76.14)
Negative perceptions	2 (2.27)	3 (3.41)	4 (4.55)	6 (6.82)	7 (7.95)
Varying perceptions	1 (1.14)	5 (5.68)	5 (5.68)	12 (13.64)	11 (12.5)
Undetermined	2 (2.27)	2 (2.27)	2 (2.27)	5 (5.68)	3 (3.41)
**Are the recommendations feasible to implement?**					
	**Permanent Supportive Housing** **n (%)**	**Income Assistance** **n (%)**	**Case Management** **n (%)**	**Supervised Consumption Facilities** **n (%)**	**Opioid Agonist Therapy** **n (%)**
Positive perceptions	76 (86.36)	76 (86.36)	60 (68.18)	68 (77.27)	66 (75)
Negative perceptions	3 (3.41)	2 (2.27)	12 (13.64)	5 (5.68)	5 (5.68)
Varying perceptions	7 (7.95)	5 (5.68)	8 (9.09)	5 (5.68)	5 (5.68)
Undetermined	2 (2.27)	5 (5.68)	8 (9.09)	10 (11.36)	12 (13.64)
**Are the recommendations acceptable to stakeholders?**					
	**Permanent Supportive Housing** **n (%)**	**Income Assistance** **n (%)**	**Case Management** **n (%)**	**Supervised Consumption Facilities** **n (%)**	**Opioid Agonist Therapy** **n (%)**
Positive perceptions	74 (84.09)	75 (85.23)	70 (79.55)	69 (78.41)	74 (84.09)
Negative perceptions	3 (3.41)	2 (2.27)	7 (7.95)	3 (3.41)	1 (1.14)
Varying perceptions	4 (4.55)	4 (4.55)	1 (1.14)	2 (2.27)	2 (2.27)
Undetermined	7 (7.95)	7 (7.95)	10 (11.36)	14 (15.90)	11 (12.5)
**How large are the costs of implementing the recommendations?**					
	**Permanent Supportive Housing** **n (%)**	**Income Assistance** **n (%)**	**Case Management** **n (%)**	**Supervised Consumption Facilities** **n (%)**	**Opioid Agonist Therapy** **n (%)**
Moderate to large costs	27 (30.68)	20 (22.73)	35 (39.77)	8 (9.09)	26 (29.54)
Negligible effect	10 (11.36)	23 (26.14)	6 (6.82)	3 (3.41)	9 (10.23)
Moderate to large savings	27 (30.68)	19 (21.59)	21 (23.86)	60 (68.18)	24 (27.27)
Varying perceptions	6 (6.82)	8 (9.09)	6 (6.82)	7 (7.95)	9 (10.23)
Undetermined	18 (20.45)	18 (20.45)	20 (22.73)	10 (11.36)	20 (22.73)
**What would be the impact of the recommendations on health equity?**					
	**Permanent Supportive Housing** **n (%)**	**Income Assistance** **n (%)**	**Case Management** **n (%)**	**Supervised Consumption Facilities** **n (%)**	**Opioid Agonist Therapy** **n (%)**
Positive impact	70 (79.55)	67 (76.14)	65 (73.86)	62 (70.45)	64 (72.73)
Negative or no impact	10 (11.36)	9 (10.23)	10 (11.36)	9 (10.23)	5 (5.68)
Varying perceptions	2 (2.27)	5 (5.68)	4 (4.55)	7 (7.95)	7 (7.95)
Undetermined	6 (6.82)	7 (7.95)	9 (10.23)	10 (11.36)	12 (13.64)
**Do you intend to implement these recommendations?**					
	**Permanent Supportive Housing** **n (%)**	**Income Assistance** **n (%)**	**Case Management** **n (%)**	**Supervised Consumption Facilities** **n (%)**	**Opioid Agonist Therapy** **n (%)**
Positive perceptions	64 (72.72)	69 (78.41)	66 (75)	66 (75)	68 (77.27)
Negative perceptions	4 (4.55)	3 (3.41)	6 (6.82)	5 (5.68)	4 (4.55)
Varying perceptions	0 (0)	1 (1.14)	0 (0)	0 (0)	0 (0)
Undetermined	20 (22.73)	15 (17.04)	16 (18.18)	17 (19.32)	16 (18.18)

* Participants who responded “yes” or “probably yes” were collapsed into positive perceptions. Responses of “no” or “probably no” were combined as negative perceptions. Responses of “varies” are listed as varying perceptions and responses of “don’t know” are listed as undetermined.

## Supplementary Materials

The following are available online at https://www.mdpi.com/1660-4601/17/21/7938/s1, Additional File 1: The Consolidated criteria for reporting qualitative studies (COREQ): 32-item checklist, Additional File 2: FACE Survey, Additional File 3: Subgroup Survey Data, Additional File 4: Theoretical Domains Framework.
