# Supplementary material for: Determinants of Implementation of a Clinical Practice Guideline for Homeless Health
Source: Int J Environ Res Public Health. 2020 Oct 29;17(21):7938. doi: 10.3390/ijerph17217938 (PMC7663114; doi:10.3390/ijerph17217938)
Supplement: Supplementary file 1 [file ijerph-17-07938-s001.pdf]

Additional File 1: Consolidated criteria for reporting qualitative studies (COREQ): 32-item checklist.

No	Item	Guide questions/description	Page number
		**Domain 1: Research team and reflexivity**	
		Personal Characteristics	
1.	Interviewer / facilitator	Which author/s conducted the interview or focus group?	6,7
2.	Credentials	What were the researcher's credentials? *E.g. PhD, MD*	6,7
3.	Occupation	What was their occupation at the time of the study?	6,7
4.	Gender	Was the researcher male or female?	6,7
5.	Experience and training	What experience or training did the researcher have?	6,7
		Relationship with participants	
6.	Relationship established	Was a relationship established prior to study commencement?	6,7
7.	Participant knowledge of the interviewer	What did the participants know about the researcher? e.*g. personal goals, reasons for doing the research*	6,7
8.	Interviewer characteristics	What characteristics were reported about the interviewer/facilitator? e.g. *Bias, assumptions, reasons and interests in the research topic*	6,7
		**Domain 2: study design**	
		Theoretical framework	
9.	Methodological orientation and Theory	What methodological orientation was stated to underpin the study? *e.g. grounded theory, discourse analysis, ethnography, phenomenology, content analysis*	6,9,10
		Participant selection	
10.	Sampling	How were participants selected? *e.g. purposive, convenience, consecutive, snowball*	7
11.	Method of approach	How were participants approached? e.*g. face-to-face, telephone, mail, email*	7
12.	Sample size	How many participants were in the study?	7,10
13.	Non-participation	How many people refused to participate or dropped out? Reasons?	N/A
Setting			
14.	Setting of data collection	Where was the data collected? e.*g. home, clinic, workplace*	7
15.	Presence of non-participants	Was anyone else present besides the participants and researchers?	8,9

16.	Description of sample	What are the important characteristics of the sample? *e.g. demographic data, date*	10,11
		Data collection	
17.	Interview guide	Were questions, prompts, guides provided by the authors? Was it pilot tested?	Additional Files
18.	Repeat interviews	Were repeat interviews carried out? If yes, how many?	N/A
19.	Audio/visual recording	Did the research use audio or visual recording to collect the data?	8,9
20.	Field notes	Were field notes made during and/or after the interview or focus group?	8,9
21.	Duration	What was the duration of the interviews or focus group?	8,9
22.	Data saturation	Was data saturation discussed?	7
23.	Transcripts returned	Were transcripts returned to participants for comment and/or correction?	9
		**Domain 3: analysis and findings**	
		Data analysis	
24.	Number of data coders	How many data coders coded the data?	9,10
25.	Description of the coding tree	Did authors provide a description of the coding tree?	9,10, Additional Files
26.	Derivation of themes	Were themes identified in advance or derived from the data?	9,10
27.	Software	What software, if applicable, was used to manage the data?	N/A
28.	Participant checking	Did participants provide feedback on the findings?	10
Reporting			
29.	Quotations presented	Were participant quotations presented to illustrate the themes / findings? Was each quotation identified? e.*g. participant number*	16-24
30.	Data and findings consistent	Was there consistency between the data presented and the findings?	16-24
31.	Clarity of major themes	Were major themes clearly presented in the findings?	16-24
32.	Clarity of minor themes	Is there a description of diverse cases or discussion of minor themes?	N/A

Additional File 2: FACE Survey

Date:

City:

Participant #:

BEGIN SURVEY

Section A - Demographics:

1. My age is:
    a. Less than 30
    b. 31-40
    c. 41-50
    d. 51-60
    e. 60 or more

2. My gender is:
    a. Male
    b. Female
    c. Other

3. My first language is:
    a. English
    b. French
    c. Other (please specify): ___________

4. Please indicate the province that you work or live in: _____________

5. Please indicate your role in providing input (select the role/group that you feel you represent the most)
    a) I am or have been homeless
    b) Primary care practitioner
    c) Specialist physician
    d) Registered nurse
    e) Public health expert

f) Social worker
g) Homelessness health researcher
h) Community health advocate
i) Other (please specify):________

6. At some time in my life, I have been homeless or vulnerably housed for: (we define vulnerably housed as living in poor-quality, temporary, or precarious type of housing, including single room hotels, shelters or rooming houses)

a. Less than 2 years
b. 2-5 years
c. 6-10 years
d. 11+ years
e. Not applicable

7. I have been involved in clinical care or research related to the health of homeless or vulnerably housed people for:

a. Less than 2 years
b. 2-5 years
c. 6-10 years
d. 11+ years
e. Not applicable

Section B: Priority Setting

We would like you to identify which of the following interventions are considered a priority for homeless populations in Canada.

For each intervention below, select a response to the question "Is the intervention a priority?"

1. Permanent Supportive Housing
    a. No
    b. probably no
    c. probably yes
    d. Yes
    e. Varies
    f. don't know

2. Income Assistance
    a. No
    b. probably no
    c. probably yes
    d. Yes
    e. Varies
    f. don’t know

3. Case Management (Intensive case management, assertive community treatment, critical time intervention)
    a. No
    b. probably no
    c. probably yes
    d. Yes
    e. Varies
    f. don’t know

4. Supervised Consumption Facilities
    a. No
    b. probably no
    c. probably yes
    d. Yes
    e. Varies
    f. don’t know

5. Opioid agonist therapy
    a. No
    b. probably no
    c. probably yes
    d. Yes
    e. Varies
    f. don’t know

Section C: FACE Explanation

In the following section, we will ask you to assess the Feasibility, Acceptability, Cost and Equity (FACE) of the drafted recommendations. Please read the definitions of the GRADE FACE constructs below before proceeding to the next section.

Construct	Question	Explanation
Feasibility	Is the recommendation feasible to implement?	The less feasible (capable of being accomplished or brought about) an option is, the less likely it is that it should be recommended (i.e. the more barriers there are that would be difficult to overcome).
Acceptabilit y	Is the recommendation acceptable to stakeholders (including your organization)?	The less acceptable an option is to key stakeholders, the less likely it is that it should be recommended, or if it is recommended, the more likely it is that the recommendation should include an implementation strategy to address concerns about acceptability. Acceptability might reflect who benefits (or is harmed) and who pays (or saves); and when the benefits, adverse effects, and costs occur (and the discount rates of key stakeholders; e.g. politicians may have a high discount rate for anything that occurs beyond the next election).
Cost	How large are the costs of implementing the recommendation?	The greater the cost, the less likely it is that an option should be a priority. Conversely, the greater the savings, the more likely it is that an option should be a priority.
Equity	What would be the impact on health equity?	Policies or programmes that reduce inequities are more likely to be a priority than ones that do not (or ones that increase inequities).

Section D: Recommendations

For each of the following five conditions, please provide feedback on the feasibility, acceptability, cost and equity of the recommendations.

**Condition 1:**	A person experiencing homelessness or vulnerable housing
**Recommendation 1:**	Identify, during history and physical examination, homelessness or housing vulnerability and willingness to consider housing.
**Recommendation 2:**	Refer homeless or vulnerably housed individuals to local housing coordinator or case manager (i.e. by dialing 211 in Ontario or via a social worker) for immediate link to permanent supportive housing and coordinated assessment system (*Strong recommendation, moderate certainty evidence*).
**Implementation considerations:**	Will require partnership with the Canadian National Housing Strategy, *Reaching Home*. This partnership should be at local, provincial and federal levels.<br><br>Local context should be considered when implementing and tailoring permanent supportive housing models to meet local needs.For example, in metropolitan areas the housing subsidy may need to be higher due to tighter housing market.<br><br>Practitioners will require questions to identify people’s housing status. For example, Q1) Where did you sleep last night? Q2) How long have you stayed in the place you stayed last night? Q3) Where

	were you staying prior to the place you stayed last night? Q4) Is there violence or conflict in the place you were staying last night? Q5) Is your health or safety at risk in the place you were staying last night?

1. Are the recommendations feasible to implement?
   (Alternate wording: Is it realistically possible to put the recommendations into practice?)
   a. No
   b. Probably no
   c. probably yes
   d. Yes
   e. Varies
   f. don't know

2. Are the recommendations acceptable to stakeholders (including your organization)?
   (Alternate wording: Are the recommendations acceptable to individuals who are homeless or have been homeless in the past?)
   a. No
   b. Probably no
   c. Probably yes
   d. Yes
   e. Varies
   f. Don't know

3. How large are the costs of implementing the recommendations?
   (Alternate wording: How much would it cost to put the recommendations into practice?)
   a. Large costs
   b. moderate costs
   c. negligible costs and savings
   d. moderate savings
   e. large savings
   f. Varies
   g. don't know

4. What would be the impact of the recommendations on health equity?

(Alternate wording: Do you think there are groups of people that would be disadvantaged by the recommendations?)

a. Reduced
b. Probably reduced
c. probably no impact
d. probably increased
e. Increased
f. Varies
g. Don't know

(Alternate scale: No / probably no / probably yes / yes / varies / don't know)

5. **IF THE PARTICIPANT IS A <u>SERVICE PROVIDER</u>**:Do you intend to implement these recommendations? (Alternate wording: Do you plan to put the recommendations into practice?)
   IF THE PARTICIPANT IS A <u>SERVICE USER</u>: Do you intend to request these interventions from your primary care provider? (Alternate wording: Do you plan to ask your family doctor about getting help to access these services?)
   a. No
   b. Probably no
   c. Probably yes
   d. Yes
   e. don't know

Comments: ______________________________

**Condition 2:**	A homeless or vulnerably housed individual with experience of poverty, income instability, or living in low-income households
**Recommendation 1:**	Assess a homeless or vulnerably housed individual for income insecurity
**Recommendation 2:**	Assist individuals with income insecurity to identify and access income support resources (*Conditional recommendation, low certainty evidence*).
**Implementation considerations:**	Practitioners should watch for income instability, housing insecurity and other related social determinants of health such as disability, unemployment, or social exclusion. Consult poverty tools when needed (e.g. https://cep.health/clinical-products/poverty-a-clinical-tool-for-primary-care-providers/)<br><br>Practitioners should inform themselves of social determinants of health resources (such as social assistance programs, disability income support programs, tax benefits, or other income assistance resources e.g. 211 in Ontario).

Practitioners will require questions to identify income instability. For example: Do you ever have trouble making ends meet at the end of month?

6. Are the recommendations feasible to implement?
   (Alternate wording: Is it realistically possible to put the recommendations into practice?)
   a. No
   b. Probably no
   c. probably yes
   d. Yes
   e. Varies
   f. don't know

7. Are the recommendations acceptable to stakeholders (including your organization)?
   (Alternate wording: Are the recommendations acceptable to individuals who are homeless or have been homeless in the past?)
   a. No
   b. Probably no
   c. Probably yes
   d. Yes
   e. Varies
   f. Don't know

8. How large are the costs of implementing the recommendations?
   (Alternate wording: How much would it cost to put the recommendations into practice?)
   a. Large costs
   b. moderate costs
   c. negligible costs and savings
   d. moderate savings
   e. large savings
   f. Varies
   g. don't know

9. What would be the impact of the recommendations on health equity?

(Alternate wording: Do you think there are groups of people that would be disadvantaged by the recommendations?)

a. Reduced
b. Probably reduced
c. probably no impact
d. probably increased
e. Increased
f. Varies
g. Don't know

(Alternate scale: No / probably no / probably yes / yes / varies / don't know)

10. **IF THE PARTICIPANT IS A <u>SERVICE PROVIDER</u>**:Do you intend to implement these recommendations? (Alternate wording: Do you plan to put the recommendations into practice?)

IF THE PARTICIPANT IS A <u>SERVICE USER</u>: Do you intend to request these interventions from your primary care provider? (Alternate wording: Do you plan to ask your family doctor about getting help to access these services?)

a. No
b. Probably no
c. Probably yes
d. Yes
e. don't know

Comments: ____________________________

**Condition 3:**	A homeless or vulnerably housed individual with multiple comorbid or complex health needs (including mental illness and/or substance use)
**Recommendation 1:**	Identify history of severe mental illness, such as psychotic or mood and anxiety disorders, substance use or multiple/complex health needs.
**Recommendation 2:**	Refer to local community mental health programs, psychiatric services, or other local resources for assessment and linkage to intensive case management (ICM), assertive community treatment (ACT), or critical time interventions (CTI) where available. Otherwise, refer to comprehensive primary care with access to on site psychiatrist for assessment and connection to the most appropriate local resources (*Conditional recommendation, low certainty evidence*)
**Implementation considerations:**	Referral to these services can be facilitated by mental health specialists, and other professionals familiar with local access points. Providers should familiarize themselves with clinic and other local resources as well as intervention variability depending on jurisdiction to inform referrals (Referral to local agencies, 211 helpline)

	ACT, ICM and CTI adopt a trauma-informed strengths-based approach respectful of the capacity, skills, knowledge, connections and potential in individuals and communities. It is important that primary care providers maintain frequent contact with ACT/ICM/CTI teams to improve continuity and coordination of comprehensive services.

11. Are the recommendations feasible to implement?

    (Alternate wording: Is it realistically possible to put the recommendations into practice?)

    a. No
    b. Probably no
    c. probably yes
    d. Yes
    e. Varies
    f. don’t know

12. Are the recommendations acceptable to stakeholders (including your organization)?

    (Alternate wording: Are the recommendations acceptable to individuals who are homeless or have been homeless in the past?)

    a. No
    b. Probably no
    c. Probably yes
    d. Yes
    e. Varies
    f. Don’t know

13. How large are the costs of implementing the recommendations?

    (Alternate wording: How much would it cost to put the recommendations into practice?)

    a. Large costs
    b. moderate costs
    c. negligible costs and savings
    d. moderate savings
    e. large savings
    f. Varies
    g. don’t know

14. What would be the impact of the recommendations on health equity? (Alternate wording: Do you think there are groups of people that would be disadvantaged by the recommendations?)
    a. Reduced
    b. Probably reduced
    c. probably no impact
    d. probably increased
    e. Increased
    f. Varies
    g. Don’t know
    (Alternate scale: No / probably no / probably yes / yes / varies / don’t know)

15. **IF THE PARTICIPANT IS A SERVICE PROVIDER**:Do you intend to implement these recommendations? (Alternate wording: Do you plan to put the recommendations into practice?)
    IF THE PARTICIPANT IS A SERVICE USER: Do you intend to request these interventions from your primary care provider? (Alternate wording: Do you plan to ask your family doctor about getting help to access these services?)
    a. No
    b. Probably no
    c. Probably yes
    d. Yes
    e. don’t know

Comments: ___________________________

**Condition 4:**	A homeless or vulnerably housed individual currently using opioids
**Recommendation 1:**	Identify, during history or physical examination, opioid use disorder.
**Recommendation 2:**	Ensure access within primary care or via an addiction specialist to opioid agonist therapy (OAT), potentially in collaboration with public health or community health centre for linkage to pharmacological interventions (*Conditional recommendation, low certainty evidence*).
**Implementation considerations:**	All patients on opioid medication should have a Naloxone kit at home, receive required training and pick up at an official distributor.<br><br>Close collaboration and training required between primary and specialty care providers, educators, health system, and professional associations to optimize access.

Methadone and buprenorphine prescribing is no longer restricted in all Canadian provinces. Counselling services may be required in addition to pharmacological interventions.

16. Are the recommendations feasible to implement?

    (Alternate wording: Is it realistically possible to put the recommendations into practice?)

    a. No
    b. Probably no
    c. probably yes
    d. Yes
    e. Varies
    f. don't know

17. Are the recommendations acceptable to stakeholders (including your organization)?

    (Alternate wording: Are the recommendations acceptable to individuals who are homeless or have been homeless in the past?)

    a. No
    b. Probably no
    c. Probably yes
    d. Yes
    e. Varies
    f. Don't know

18. How large are the costs of implementing the recommendations?

    (Alternate wording: How much would it cost to put the recommendations into practice?)

    a. Large costs
    b. moderate costs
    c. negligible costs and savings
    d. moderate savings
    e. large savings
    f. Varies
    g. don't know

19. What would be the impact of the recommendations on health equity? (Alternate wording: Do you think there are groups of people that would be disadvantaged by the recommendations?)
    a. Reduced
    b. Probably reduced
    c. probably no impact
    d. probably increased
    e. Increased
    f. Varies
    g. Don't know

    (Alternate scale: No / probably no / probably yes / yes / varies / don't know)

20. **IF THE PARTICIPANT IS A SERVICE PROVIDER**:Do you intend to implement these recommendations? (Alternate wording: Do you plan to put the recommendations into practice?)
    IF THE PARTICIPANT IS A SERVICE USER: Do you intend to request these interventions from your primary care provider? (Alternate wording: Do you plan to ask your family doctor about getting help to access these services?)
    a. No
    b. Probably no
    c. Probably yes
    d. Yes
    e. don't know

Comments: ____________________________

**Condition 5:**	A homeless or vulnerably housed individual with substance use
**Recommendation 1:**	Identify, during history or physical examination, problematic substance use including alcohol or other drugs.
**Recommendation 2:**	Identify the most appropriate approach or refer to local addiction and harm reduction/prevention services (e.g. supervised consumption facilities, managed alcohol programs) via appropriate local resources such as public health or community health centre/CLSC (*Conditional recommendation, low certainty evidence*).
**Recommendation 3:**	In case of active opioid use disorder, ensure access within primary care or via an addictions specialist to opioid agonist therapy.
**Implementation considerations:**	Practitioners need to identify needs and goals of the individual person and then to refer to the most appropriate service. Practitioners and patients should be aware of the closest supervised consumption facility location and hours of operation.

Incorporate trauma-informed training and expertise in substance use treatment for primary care to build trust between patient and practitioner.

21. Are the recommendations feasible to implement?
    (Alternate wording: Is it realistically possible to put the recommendations into practice?)
    a. No
    b. Probably no
    c. probably yes
    d. Yes
    e. Varies
    f. don't know

22. Are the recommendations acceptable to stakeholders (including your organization)?
    (Alternate wording: Are the recommendations acceptable to individuals who are homeless or have been homeless in the past?)
    a. No
    b. Probably no
    c. Probably yes
    d. Yes
    e. Varies
    f. Don't know

23. How large are the costs of implementing the recommendations?
    (Alternate wording: How much would it cost to put the recommendations into practice?)
    a. Large costs
    b. moderate costs
    c. negligible costs and savings
    d. moderate savings
    e. large savings
    f. Varies
    g. don't know

24. What would be the impact of the recommendations on health equity?

(Alternate wording: Do you think there are groups of people that would be disadvantaged by the recommendations?)

a. Reduced
b. Probably reduced
c. probably no impact
d. probably increased
e. Increased
f. Varies
g. Don't know

(Alternate scale: No / probably no / probably yes / yes / varies / don't know)

25. **IF THE PARTICIPANT IS A __SERVICE PROVIDER__**:Do you intend to implement these recommendations? (Alternate wording: Do you plan to put the recommendations into practice?)

IF THE PARTICIPANT IS A __SERVICE USER__: Do you intend to request these interventions from your primary care provider? (Alternate wording: Do you plan to ask your family doctor about getting help to access these services?)

a. No
b. Probably no
c. Probably yes
d. Yes
e. don't know

Comments: ____________________________

__Section E: Follow-up questions__

1. Our recommendations highlight the important role of permanent supportive housing. Please tell us your experience with housing services in your area.

2. We recognize that our health and social systems can sometimes leave gaps in services. Please tell us your experience with case management or care coordination services.

3. Please tell us your experience with opioid maintenance therapy or other substance use services.

4. Is there anything else you would like to share on these topics?

Additional File 3: Subgroup Survey Data

Demographics

Characteristic	Health and Social Service Providers (n = 74)	Lived Experience (n = 14)	Total n = 88 (%)
**Age**			
< 30 years	**13 (17.6)**	**1 (7.14)**	**14 (15.9)**
31-40 years	**20 (27.0)**	**4 (28.6)**	**24 (27.3)**
41-50 years	**19 (25.7)**	**4 (28.6)**	**23 (26.1)**
51-60 years	**12 (16.2)**	**2 (14.3)**	**14 (15.9)**
61+ years	**8 (10.8)**	**3 (21.4)**	**11 (12.5)**
Missing	**2 (2.70)**	**0 (0.00)**	**2 (2.27)**
**Gender**			
Male	**30 (40.5)**	**8 (57.1)**	**38 (43.2)**
Female	**43 (58.1)**	**6 (42.9)**	**49 (55.7)**
Other	**0 (0.00)**	**0 (0.00)**	**0 (0.00)**
Missing	**1 (1.35)**	**0 (0.00)**	**1 (1.14)**
**Province**			
British Columbia	**5 (6.76)**	**0 (0.00)**	**5 (5.68)**
Alberta	**16 (21.6)**	**0 (0.00)**	**16 (18.2)**
Ontario	**42 (56.8)**	**11 (78.6)**	**53 (60.2)**
Quebec	**9 (12.2)**	**3 (21.4)**	**12 (13.6)**
Nova Scotia	**1 (1.35)**	**0 (0.00)**	**1 (1.14)**
Prince Edward Island	**1 (1.35)**	**0 (0.00)**	**1 (1.14)**
Missing	**0 (0.00)**	**0 (0.00)**	**0 (0.00)**
**First language**			
English	**63 (85.1)**	**11 (78.6)**	**74 (84.1)**
French	**5 (6.76)**	**2 (14.3)**	**7 (7.95)**
Other	**4 (5.41)**	**1 (7.14)**	**5 (5.68)**
Missing	**2 (2.70)**	**0 (0.00)**	**2 (2.27)**
**Profession**			
Primary care provider	**32 (43.2)**	**0 (0.00)**	**32 (36.4)**

Specialist physician	**10 (13.5)**	**0 (0.00)**	**10 (11.4)**
Registered nurse	**4 (5.41)**	**0 (0.00)**	**4 (4.55)**
Public health expert	**1 (1.35)**	**0 (0.00)**	**1 (1.14)**
Social worker	**1 (1.35)**	**0 (0.00)**	**1 (1.14)**
Homelessness health researcher	**10 (13.5)**	**0 (0.00)**	**10 (11.4)**
Community health advocate	**0 (0.00)**	**0 (0.00)**	**0 (0.00)**
I am or have been homeless	**2 (2.70)**	**14 (100)**	**16 (18.2)**
Other++	**13 (17.6)**	**0 (0.00)**	**13 (14.8)**
Missing	**1 (1.35)**	**0 (0.00)**	**1 (1.14)**
**Length of homelessness experience[*]**			
< 2 years	**9 (12.2)**	**2 (14.3)**	**11 (12.5)**
2-5 years	**1 (1.35)**	**5 (35.7)**	**6 (6.82)**
6-10 years	**1 (1.35)**	**1 (7.14)**	**2 (2.27)**
11+ years	**0 (0.00)**	**5 (35.7)**	**5 (5.68)**
Not applicable	**63 (85.1)**	**1 (7.14)**	**64 (72.7)**
Missing	**0 (0.00)**	**0 (0.00)**	**0 (0.00)**
**Length of involvement in homelessness research or programs**			
< 2 years	**12 (16.2)**	**0 (0.00)**	**12 (13.6)**
2-5 years	**9 (12.2)**	**1 (7.14)**	**10 (11.4)**
6-10 years	**19 (25.7)**	**0 (0.00)**	**19 (21.6)**
11+ years	**25 (33.8)**	**0 (0.00)**	**25 (28.4)**
Not applicable	**9 (12.2)**	**13 (92.9)**	**22 (25.0)**
Missing	**0 (0.00)**	**0 (0.00)**	**0 (0.00)**

Priority setting - Health and Social Service Providers, N = 74

	**Permanent supportive housing**	**Income assistance**	**Case management***	**Supervised consumption facilities**	**Opioid agonist therapy**
Is the intervention a priority?					
No	0 (0.00)	1 (1.35)	0 (0.00)	0 (0.00)	1 (1.35)
Probably no	1 (1.35)	0 (0.00)	3 (4.05)	3 (4.05)	2 (2.70)
Probably yes	3 (4.05)	9 (12.2_	17 (23.0)	14 (18.9)	17 23.0)

Yes	67 (90.5)	59 (79.7)	49 (66.2)	40 (54.1)	41 (55.4)
Varies	1 (1.35)	3 (4.05)	3 (4.05)	12 (16.2)	11 (14.9)
Don't know	1 (1.35)	1 (1.35)	1 (1.35)	4 (5.41)	1 (1.35)
Missing	1 (1.35)	1 (1.35)	1 (1.35)	1 (1.35)	1 (1.35)

Priority setting - Lived Experience Stakeholders, N = 14.

	**Permanent supportive housing**	**Income assistance**	**Case management***	**Supervised consumptio n facilities**	**Opioid agonist therapy**
Is the intervention a priority?					
No	1 (7.14)	2 (14.3)	0 (0.00)	3 (21.4)	4 (28.6)
Probably no	0 (0.00)	0 (0.00)	1 (7.14)	0 (0.00)	0 (0.00)
Probably yes	1 (7.14)	1 (7.14)	0 (0.00)	0 (0.00)	0 (0.00)
Yes	12 85.7)	9 (64.3)	11 (78.6)	11 (78.6)	9 (64.3)
Varies	0 (0.00)	2 (14.8)	2 (14.3)	0 (0.00)	0 (0.00)
Don't know	0 (0.00)	0 (0.00)	0 (0.00)	0 (0.00)	1 (7.14)
Missing	0 (0.00)	0 (0.00)	0 (0.00)	0 (0.00)	0 (0.00)

*Intensive case management, assertive community treatment, critical time intervention.

Priority setting - All Stakeholders, N = 88

	**Permanent supportive housing**	**Income assistance**	**Case management***	**Supervised consumption facilities**	**Opioid agonist therapy**
Is the intervention a priority?					
No	1 (1.14)	3 (3.41)	0 (0.00)	3 (3.41)	5 (5.68)
Probably no	1 (1.14)	0 (0.00)	4 (4.55)	3 (3.41)	2 (2.27)
Probably yes	4 (4.55)	10 (11.4)	17 (19.3)	14 (15.9)	17 (19.3)
Yes	79 (90.0)	68 (77.3)	60 (68.2)	51 (58.0)	50 (56.8)
Varies	1 (1.14)	5 (5.68)	5 (5.68)	12 (13.6)	11 (12.5)
Don't know	1 (1.14)	1 (1.14)	1 (1.14)	4 (4.55)	2 (2.27)
Missing	1 (1.14)	1 (1.14)	1 (1.14)	1 (1.14)	1 (1.14)

*Intensive case management, assertive community treatment, critical time intervention.

FACE constructs: Health and Social Service Providers, N = 74

**FACE**	**Permanent Supportive Housing**	**Income Assistance**	**Case Management**	**Supervised Consumption Facility**	**Opioid Agonist Therapy**
Are the recommendations feasible to implement?					
Yes	33 (44.6)	45 (60.8)	24 (32.4)	34 (46.0)	36 (48.6)
Probably yes	31 (41.9)	19 (25.7)	25 (33.8)	23 (31.1)	20 (27.0)
Probably no	1 (1.35)	2 (2.70)	7 (9.46)	4 (5.41)	3 (4.05)
No	1 (1.35)	0 (0.00)	3 (4.05)	1 (1.35)	1 (1.35)
Varies	6 (8.11)	3 (4.05)	7 (9.46)	3 (4.05)	3 (4.05)
Don't Know	0 (0.00)	1 (1.35)	3 (4.05)	3 (4.05)	5 (6.76)
Missing	2 (2.70)	4 (5.41)	5 (6.76)	6 (8.11)	6 (8.11)
Are the recommendations					

acceptable to stakeholders?					
Yes	47 (63.5)	42 (56.8)	41 (55.4)	39 (52.7)	34 (46.0)
Probably yes	18 (24.3)	21 (28.4)	17 (23.0)	20 (27.0)	29 (39.2)
Probably no	2 (2.70)	2 (2.70)	4 (5.41)	1 (1.35)	1 (1.35)
No	0 (0.00)	0 (0.00)	2 (2.70)	1 (1.35)	0 (0.00)
Varies	0 (0.00)	0 (0.00)	0 (0.00)	0 (0.00)	0 (0.00)
Don’t know	4 (5.41)	5 (6.76)	5 (6.76)	7 (9.46)	4 (5.41)
Missing	3 (4.05)	4 (5.41)	5 (6.76)	6 (8.11)	6 (8.11)
How large are the costs of implementing the recommendations ?					
Large costs	8 (10.8)	4 (5.41)	8 (10.8)	2 (2.70)	4 (5.41)
Moderate costs	17 (23.0)	12 (16.2)	23 (31.1)	21 (28.4)	19 (25.7)
Negligible costs and savings	10 (13.5)	23 (31.1)	6 (8.11)	12 (16.2)	9 (12.2)
Moderate savings	13 (17.6)	6 (8.11)	9 (12.2)	12 (16.2)	14 (18.9)
Large savings	5 (6.76)	5 (6.76)	3 (4.05)	7 (9.46)	3 (4.05)
Varies	3 (4.05)	6 (8.11)	5 (6.76)	5 (6.76)	7 (9.46)
Don’t know	15 (20.3)	14 (18.9)	15 (20.3)	9 (12.2)	12 (16.2)
Missing	3 (4.05)	4 (5.41)	5 (6.76)	6 (8.11)	6 (8.11)
What would be the impact of the recommendations on health equity?					
Reduced	0 (0.00)	4 (5.41)	1 (1.35)	1 (1.35)	1 (1.35)
Probably reduced	4 (5.41)	2 (2.70)	4 (5.41)	3 (4.05)	3 (4.05)
Probably no impact	1 (1.35)	0 (0.00)	3 (4.05)	3 (4.05)	0 (0.00)
Probably increased	29 (39.2)	27 (36.5)	20 (27.0)	20 (27.0)	21 (28.4)
Increased	33 (44.6)	32 (43.2)	35 (47.3)	32 (43.2)	34 (45.9)
Varies	1 (1.35)	2 (2.70)	2 (2.70)	6 (8.11)	5 (6.76)
Don’t know	3 (4.05)	3 (4.05)	4 (5.41)	3 (4.05)	4 (5.41)
Missing	3 (4.05)	4 (5.41)	5 (6.76)	6 (8.11)	6 (8.11)
Do you intend to implement these recommendations ?					
Yes	42 (56.8)	43 (58.1)	32 (43.2)	38 (51.4)	40 (54.1)
Probably yes	11 (14.9)	15 (20.3)	21 (28.4)	16 (21.6)	16 (21.6)
Probably no	2 (2.70)	1 (1.35)	4 (5.41)	2 (2.70)	2 (2.70)
No	0 (0.00)	1 (1.35)	1 (1.35)	2 (2.70)	1 (1.35)
Varies	0 (0.00)	0 (0.00)	0 (0.00)	0 (0.00)	0 (0.00)
Don’t know	15 (20.3)	9 (12.2)	10 (13.5)	9 (12.2)	8 (10.8)
Missing	4 (5.41)	5 (6.76)	6 (8.11)	7 (9.46)	7 (9.46)

FACE constructs: Lived Experience, N = 14

FACE	Permanent Supportive Housing	Income Assistance	Case Management	Supervised Consumption Facility	Opioid Agonist Therapy
Are the recommendations feasible to implement?					
Yes	9 (64.3)	11 (78.6)	10 (71.4)	11 (78.6)	9 (64.3)
Probably yes	3 (21.4)	1 (7.14)	1 (7.14)	0 (0.00)	1 (7.14)

Probably no	0 (0.00)	0 (0.00)	0 (0.00)	0 (0.00)	0 (0.00)
No	1 (7.14)	0 (0.00)	2 (14.3)	0 (0.00)	1 (7.14)
Varies	1 (7.14)	2 (14.3)	1 (7.14)	2 (14.3)	2 (14.3)
Don’t Know	0 (0.00)	0 (0.00)	0 (0.00)	0 (0.00)	0 (0.00)
Missing	0 (0.00)	0 (0.00)	0 (0.00)	1 (7.14)	1 (7.14)
Are the recommendations acceptable to stakeholders?					
Yes	9 (64.3)	12 (85.7)	11 (78.6)	10 (71.4)	10 (71.4)
Probably yes	0 (0.00)	0 (0.00)	1 (7.14)	0 (0.00)	1 (7.14)
Probably no	0 (0.00)	0 (0.00)	0 (0.00)	0 (0.00)	0 (0.00)
No	1 (7.14)	0 (0.00)	1 (7.14)	1 (7.14)	0 (0.00)
Varies	4 (28.6)	2 (14.3)	1 (7.14)	2 (14.2)	2 (14.2)
Don’t know	0 (0.00)	0 (0.00)	0 (0.00)	0 (0.00)	0 (0.00)
Missing	0 (0.00)	0 (0.00)	0 (0.00)	1 (7.14)	1 (7.14)
How large are the costs of implementing the recommendations ?					
Large costs	2 (14.3)	2 (14.3)	2 (14.3)	3 (21.4)	2 (14.3)
Moderate costs	0 (0.00)	2 (14.3)	2 (14.3)	1 (7.14)	1 (7.14)
Negligible costs and savings	0 (0.00)	0 (0.00)	0 (0.00)	0 (0.00)	0 (0.00)
Moderate savings	1 (7.14)	0 (0.00)	2 (14.3)	2 (14.3)	1 (7.14)
Large savings	8 (57.8)	8 (57.8)	7 (50.0)	6 (42.9)	6 42.9)
Varies	3 (21.4)	2 (14.3)	1 (7.14)	1 (7.14)	2 (14.3)
Don’t know	0 (0.00)	0 (0.00)	0 (0.00)	0 (0.00)	1 (7.14)
Missing	0 (0.00)	0 (0.00)	0 (0.00)	1 (7.14)	1 (7.14)
What would be the impact of the recommendations on health equity?					
Reduced	3 (21.4)	1 (7.14)	1 (7.14)	0 (0.00)	1 (7.14)
Probably reduced	1 (7.14)	2 (14.3)	0 (0.00)	1 (7.14)	0 (0.00)
Probably no impact	1 (7.14)	0 (0.00)	1 (7.14)	1 (7.14)	0 (0.00)
Probably increased	1 (7.14)	1 (7.14)	2 (14.3)	1 (7.14)	0 (0.00)
Increased	7 (50.0)	7 (50.0)	8 (57.8)	9 (64.3)	9 (64.3)
Varies	1 (7.14)	3 (21.4)	2 (14.3)	1 (7.14)	2 (14.3)
Don’t know	0 (0.00)	0 (0.00)	0 (0.00)	0 (0.00)	1 (7.14)
Missing	0 (0.00)	0 (0.00)	0 (0.00)	1 (7.14)	1 (7.14)
Do you intend to implement these recommendations ?					
Yes	10 (71.4)	10 (71.4)	11 (78.6)	12 (85.7)	10 (71.4)
Probably yes	1 (7.14)	1 (7.14)	2 (14.3)	0 (0.00)	2 (14.3)
Probably no	0 (0.00)	0 (0.00)	0 (0.00)	0 (0.00)	0 (0.00)
No	2 (14.3)	1 (7.14)	1 (7.14)	1 (7.14)	1 (7.14)
Varies	0 (0.00)	0 (0.00)	0 (0.00)	0 (0.00)	0 (0.00)
Don’t know	1 (7.14)	1 (7.14)	0 (0.00)	0 (0.00)	0 (0.00)
Missing	0 (0.00)	1 (7.14)	0 (0.00)	1 (7.14)	1 (7.14)

FACE constructs: All Stakeholders, N = 88

FACE	Permanent Supportive Housing	Income Assistance	Case Management	Supervised Consumption Facility	Opioid Agonist Therapy
Are the recommendations feasible to implement?					
Yes	42 (47.7)	56 (63.6)	34 (38.6)	45 (51.1)	45 (51.1)
Probably yes	34 (38.6)	20 (22.7)	26 (29.5)	23 (26.1)	21 (23.9)
Probably no	1 (1.14)	2 (2.27)	7 (7.95)	4 (4.54)	3 (3.41)
No	2 (2.27)	0 (0.00)	5 (5.68)	1 (1.14)	2 (2.27)
Varies	7 (7.95)	5 (5.68)	8 (9.09)	5 (5.68)	5 (5.68)
Don't Know	0 (0.00)	1 (1.14)	3 (3.41)	3 (3.41)	5 (5.68)
Missing	2 (2.27)	4 (4.54)	5 (5.68)	7 (7.95)	7 (7.95)
Are the recommendations acceptable to stakeholders?					
Yes	56 (63.6)	54 (61.4)	52 (59.1)	49 (55.7)	44 (50.0)
Probably yes	18 (20.5)	21 (23.9)	18 (20.5)	20 (22.7)	30 (34.1)
Probably no	2 (2.27)	2 (2.27)	4 (4.54)	1 (1.14)	1 (1.14)
No	1 (1.14)	0 (0.00)	3 (3.41)	2 (2.27)	0 (0.00)
Varies	4 (4.54)	4 (4.54)	1 (1.14)	2 (2.27)	2 (2.27)
Don't know	4 (4.54)	5 (5.68)	5 (5.68)	7 (7.95)	4 (4.54)
Missing	3 (3.41)	4 (4.54)	5 (5.68)	7 (7.95)	7 (7.95)
How large are the costs of implementing the recommendations?					
Large costs	10 (11.4)	6 (6.82)	10 (11.4)	4 (4.54)	6 (6.82)
Moderate costs	17 (19.3)	14 (15.9)	25 (28.4)	4 (4.54)	20 (22.7)
Negligible costs and savings	10 (11.4)	23 (26.1)	6 (6.82)	3 (3.41)	9 (10.2)
Moderate savings	14 (15.9)	6 (6.82)	11 (12.5)	22 (25.0)	15 (17.0)
Large savings	13 (14.8)	13 (14.8)	10 (11.4)	38 (43.2)	9 (10.2)
Varies	6 (6.82)	8 (9.09)	6 (6.82)	7 (7.95)	9 (10.2)
Don't know	15 (17.0)	14 (15.9)	15 (17.0)	3 (3.41)	13 (14.8)
Missing	3 (3.41)	4 (4.54)	5 (5.68)	7 (7.95)	7 (7.95)
What would be the impact of the recommendations on health equity?					
Reduced	3 (3.41)	5 (5.68)	2 (2.27)	1 (1.14)	2 (2.27)
Probably reduced	5 (5.68)	4 (4.54)	4 (4.54)	4 (4.54)	3 (3.41)
Probably no impact	2 (2.27)	0 (0.00)	4 (4.54)	4 (4.54)	0 (0.00)
Probably increased	30 (34.1)	28 (31.8)	22 (25.0)	21 (23.9)	21 (23.9)
Increased	40 (45.5)	39 (44.3)	43 (48.9)	41 (46.6)	43 (48.9)
Varies	2 (2.27)	5 (5.68)	4 (4.54)	7 (7.95)	7 (7.95)
Don't know	3 (3.41)	3 (3.41)	4 (4.54)	3 (3.41)	5 (5.68)
Missing	3 (3.41)	4 (4.54)	5 (5.68)	7 (7.95)	7 (7.95)
Do you intend to implement these recommendations?					
Yes	52 (59.1)	53 (60.2)	43 (48.9)	50 (56.8)	50 (56.8)
Probably yes	12 (13.6)	16 (18.2)	23 (26.1)	16 (18.2)	18 (20.5)
Probably no	2 (2.27)	1 (1.14)	4 (4.54)	2 (2.27)	2 (2.27)

No	2 (2.27)	2 (2.27)	2 (2.27)	3 (3.41)	2 (2.27)
Varies	0 (0.00)	1 (1.14)	0 (0.00)	0 (0.00)	0 (0.00)
Don't know	16 (18.2)	10 (11.4)	10 (11.4)	9 (10.2)	8 (9.09)
Missing	4 (4.54)	6 (6.82)	6 (6.82)	8 (9.09)	8 (9.09)

Additional File 4: Theoretical Domains Framework

Domain (definition[1])	Constructs
1. Knowledge (An awareness of the existence of something)	Knowledge (including knowledge of condition /scientific rationale)
	Procedural knowledge
	Knowledge of task environment
2. Skills (An ability or proficiency acquired through practice)	Skills
	Skills development
	Competence
	Ability
	Interpersonal skills
	Practice
	Skill assessment
3. Social/Professional Role and Identity (A coherent set of behaviours and displayed personal qualities of an individual in a social or work setting)	Professional identity
	Professional role
	Social identity
	Identity
	Professional boundaries
	Professional confidence
	Group identity
	Leadership
	Organisational commitment
4. Beliefs about Capabilities (Acceptance of the truth, reality, or validity about an ability, talent, or facility that a person can put to constructive use)	Self-confidence
	Perceived competence
	Self-efficacy
	Perceived behavioural control
	Beliefs
	Self-esteem
	Empowerment

	Professional confidence
5. Optimism (The confidence that things will happen for the best or that desired goals will be attained)	Optimism
	Pessimism
	Unrealistic optimism
	Identity
6. Beliefs about Consequences (Acceptance of the truth, reality, or validity about outcomes of a behaviour in a given situation)	Beliefs
	Outcome expectancies
	Characteristics of outcome expectancies
	Anticipated regret
	Consequents
7. Reinforcement (Increasing the probability of a response by arranging a dependent relationship, or contingency, between the response and a given stimulus)	Rewards (proximal / distal, valued / not valued, probable / improbable)
	Incentives
	Punishment
	Consequents
	Reinforcement
	Contingencies
	Sanctions
8. Intentions (A conscious decision to perform a behaviour or a resolve to act in a certain way)	Stability of intentions
	Stages of change model
	Transtheoretical model and stages of change
9. Goals (Mental representations of outcomes or end states that an individual wants to achieve)	Goals (distal / proximal)
	Goal priority
	Goal / target setting
	Goals (autonomous / controlled)
	Action planning
	Implementation intention
10. Memory, Attention and Decision Processes (The ability to retain information, focus selectively on aspects of the environment and choose between two or more alternatives)	Memory
	Attention
	Attention control
	Decision making
	Cognitive overload / tiredness

11. Environmental Context and Resources<br>(Any circumstance of a person's situation or environment that discourages or encourages the development of skills and abilities, independence, social competence, and adaptive behaviour)	Environmental stressors
	Resources / material resources
	Organisational culture /climate
	Salient events / critical incidents
	Person x environment interaction
	Barriers and facilitators
12. Social influences<br>(Those interpersonal processes that can cause individuals to change their thoughts, feelings, or behaviours)	Social pressure
	Social norms
	Group conformity
	Social comparisons
	Group norms
	Social support
	Power
	Intergroup conflict
	Alienation
	Group identity
	Modelling
13. Emotion<br>(A complex reaction pattern, involving experiential, behavioural, and physiological elements, by which the individual attempts to deal with a personally significant matter or event)	Fear
	Anxiety
	Affect
	Stress
	Depression
	Positive / negative affect
	Burn-out
14. Behavioural Regulation<br>(Anything aimed at managing or changing objectively observed or measured actions)	Self-monitoring
	Breaking habit
	Action planning

1. All definitions are based on definitions from the American Psychological Associations' Dictionary of Psychology

Cane J, O'Connor D, Michie S. Validation of the theoretical domains framework for use in behaviour change and implementation research. Implement Sci. 2012;7:37. doi:10.1186/1748-5908-7-37.

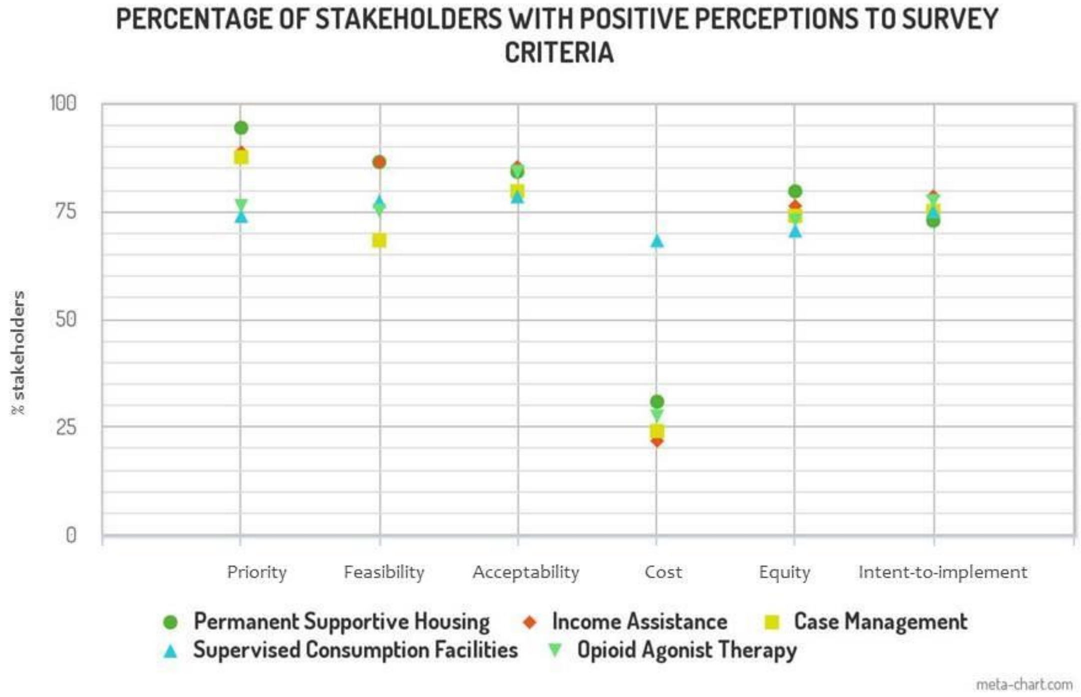

Figure 1. Perception trends.

TDF Domain	Priority	Feasibility	Acceptability	Cost	Equity	Intent
Knowledge					★	
Skills						
Social/ Professional Identity						★
Beliefs About Capability						
Optimism/ Pessimism						
Beliefs About Consequences						
Reinforcement						
Intentions						
Goals						
Memory, Attention, and Decision Procedure						
Environmental Context and Resources		★				
Social Influences			★			
Emotion/ Personal Factors						
Behavioural Regulation						
Other						

Figure 2. Heat Map.
